# Utilization of an OLED-Based VLC System in Office, Corridor, and Semi-Open Corridor Environments

**DOI:** 10.3390/s20236869

**Published:** 2020-12-01

**Authors:** Zahra Nazari Chaleshtori, Zabih Ghassemlooy, Hossien B. Eldeeb, Murat Uysal, Stanislav Zvanovec

**Affiliations:** 1 Department of Electromagnetic Field, Faculty of Electrical Engineering, Czech Technical University in Prague, 16627 Prague, Czech Republic; xzvanove@fel.cvut.cz; 2 Optical Communications Research Group, Faculty of Engineering and Environment, Northumbria University, Newcastle-upon-Tyne NE1 8ST, UK; z.ghassemlooy@northumbria.ac.uk; 3 Department of Electrical and Electronics Engineering, Ozyegin University, 34794 Istanbul, Turkey; hossien.eldeeb@ozu.edu.tr (H.B.E.); murat.uysal@ozyegin.edu.tr (M.U.)

**Keywords:** flexible OLED, visible light communications, optical path loss, delay spread

## Abstract

Organic light emitting diodes (OLEDs) have recently received growing interest for their merits as soft light and large panels at a low cost for the use in public places such as airports, shopping centers, offices, and train or bus stations. Moreover, the flexible substrate-based OLEDs provide an attractive feature of having curved or rolled lighting sources for the use in wearable devices and display panels. This technology can be implemented in visible light communications (VLC) for several applications such as visual display, data communications, and indoor localization. This article aims to investigate the use of flexible OLED-based VLC in indoor environments (i.e., office, corridor and semi-open corridor in shopping malls). We derive a two-term power series model to be match with the root-mean-square delay spread and optical path loss (OPL). We show that, for OLED positioned on outer-wall of shops, the channel gain is enhanced in contrast to them being positioned on the inner-wall. Moreover, the channel gain in empty environments is higher compare with the furnished rooms. We show that, the OPL for a 10 m link span are lower by 4.4 and 6.1 dB for the empty and semi-open corridors compared with the furnished rooms, when OLED is positioned on outer-wall of shops. Moreover, the channel gain in the corridor is higher compared with the semi-open corridor. We also show that, in furnished and semi-open corridors the OPL values are 55.6 and 57.2 dB at the center of corridor increasing to 87.6 and 90.7 dB at 20 m, respectively, when OLED is positioned on outer-wall of shops.

## 1. Introduction

Visible light communications (VLC) with its huge available bandwidth [1] and its dual functionality, i.e., illumination and safe and low-cost communications [2], has a great potential for different high data rate fixed and mobile applications [3]. The VLC technology has many advantages include inherent security, energy efficiency, healthy for human, and unregulated bandwidth [4,5]. These features make it attractive for numerous applications in different fields including indoor networking [6], vehicular communication [7], medical applications [8], Internet access for vehicles and in airplane cabins [9], and positioning systems [10].

At the receiver (Rx) side, a single photodetector (PD) is usually used, which converts the incident optical power to the electrical power. To enhance the performance, the single PD can be replaced by an angle diversity Rx, which contains multiple PDs oriented in different directions as reported in [11]. For further improvement, the field of view angles of these PDs can be optimized to increase the received signal-to-noise-ratio (SNR) as reported in [12,13]. Optical cameras have been recently introduced to capture data [14,15], resulting in interesting VLC systems applications. It is also possible to utilize the organic PDs with more synthetic flexibility [16], however the limited spectral responsivity range is the drawback. VLC relies on the use of light-emitting diodes (LEDs), organic LEDs (OLEDs) as well as white laser diodes (LDs) as the light source [17]. Owing to OLEDs attractive advantages, including transparent displays, rich color, low power consumption and large active areas [18,19], there has been a growing interest in using OLEDs for soft lighting and display applications in public places [20]. The main differences between OLEDs and the LEDs are (i) the modulation bandwidth of OLEDs, which increases linearly with the drive current, is lower than silicone LEDs (i.e., kHz compared to MHz); and (ii) OLEDs have wider radiation patterns compared with non-organic LEDs, which influences the optical path loss (OPL). This work emphasizes on utilizing OLEDs for VLC systems, where is the potential of flexible light sources could be used in office and public indoor environments.

Numerous efforts have been made in modeling VLC channel in order to determine the channel impulse response (CIR) and its characteristics in terms of the average OPL and the root-mean-square (RMS) delay spread. The achievable SNR for a given transmit power can be calculated by OPL obtained from CIR [21]. In addition, the RMS delay spread provides a good estimate of how susceptible the channel is to inter-symbol interference (ISI), thus leading to transmission data rate *R_b_* restriction [18,21]. That is why quantifying channel characteristics is vital; hence, a number of studies have been done so far. For instance, in [22], the CIR of an empty room was evaluated using Monte Carlo (MC) ray tracing at the visible wavelength range where the surface materials reflectance were not wavelength-dependent. However, in [23] the VLC channel was investigated including wavelength-dependent reflectance of materials. In [24], the modified MC ray tracing approach was used for analyzing the CIR as a function of the wavelength using a simplified matrix model. In [25], a three-dimensional (3D) model based on MC algorithm using a CAD software was presented for the VLC system. A simulation of VLC channel by the use of OpticStudio^®^ simulator produced by Zemax [26] was reported in [27,28], which was endorsed by the IEEE 802.15.7r1 Task Group. In addition, the use of OpticStudio^®^ for validation of the channel modelling was reported in [29]. Recently, utilizing OLEDs in VLC systems has captured attention. In [30], it is claimed that the use of curved OLED in VLC system for an empty room offers lower RMS delay spread and the average OPL values of 8.8% and 3 dB, respectively compared with Lambertian source. The impact of reflections using flat and half-circular OLEDs in a furnished office was investigated [31]. The recorded results in [31] reveals the ability of OLED based VLC system to achieve *R_b_* of 4 Mb/s with a bit-error-rate (BER) below the forward error correction BER limit. In [32], investigating of a flexible OLED-based VLC link in a shopping mall was reported, in both empty and furnished rooms using both full and half-circular OLEDs. The results indicated that, the OPL in an empty room is about 5 dB less than the furnished room.

Currently OLED panels are more costly than LEDs; however, with advances made in fabrication and manufacturing as well as the wider use of OLED-based lights the cost will be reduced as was the case with the non-organic LEDs a few years ago. Since, OLEDs come in different shapes and size, we have decided to investigate their characteristics when used as a transmitter (Tx) in VLC systems. This work emphasizes on the evaluation of an attractive feature of OLEDs, which is the mechanically flexible potential for utilizing in VLC system. The simulation was carried out to determine the impact of the symmetrical beam pattern of curved OLEDs, which is wider than Lambertian, on the VLC channel. In this work, we consider a VLC system in a typical office, corridor, and semi-open corridor environments with and without furniture. In the office environment, the user (i.e., the Rx), is moving along a circular path while holding a mobile phone. In corridor and semi-open corridors, the user is then moving on a straight path along the corridor. We investigate the proposed system optical features and show a new numerical model for the RMS delay-spread and OPL for the channel. We provide statistics for the BER performance and compared it for curved and flat OLED-based VLC systems.

The rest of the paper is organized as follows. In Section 2, the features of simulation and scenarios are described. Section 3 discusses the results. Finally, conclusions are given in Section 4.

## 2. Simulation 

### 2.1. Simulation Features

To determine the detected optical power and path lengths from the Tx to the Rx, non-sequential ray-tracing approach was used in the 3D environment. It evolves the specification and location of the Tx and the Rx, features of the CAD models of objects, wavelength-dependent reflectance of surfaces (wall, ceiling, floor, and objects), and transmission/reflection coefficient of glass windows. Next, the captured output data of the OpticStudio^®^ is processed in MATLAB to obtain the CIR expressed as given by [27,32]:

(1) $$h(t)=\sum_{i=1}^{N}P_{i}\delta (t-\tau_{i}),$$

where *P_i_* and $\tau_{i}$ are the power and the propagation time of the *i*th ray, respectively. δ is Dirac delta function and *N* is the number of rays received at the Rx. Note, a number of reflections from the floor, ceiling, walls, and other objects are considered until the normalized intensity of rays after intercepting an object drops to 10^−3^.

The spatial intensity distribution of light emitted from the light source is determined by the optical radiation pattern profile. The luminous intensity defined in terms of the angle of irradiance ϕ is given as [1]:

(2) $$I(\phi )=\frac{m_{L}+1}{2\pi }I(0)\cos^{m_{L}}(\phi ),\;\phi =[-\frac{\pi }{2},\frac{\pi }{2}]$$

where *I*(0) is the center luminous intensity of the OLED and *m_L_* is Lambertian order, which is defined in terms of the Tx semi-angle ϕ_1/2_ as [1]:

(3) $$m_{L}=-\frac{\ln(2)}{\ln[\cos(\phi_{1/2})]}.$$

**Figure 1:**
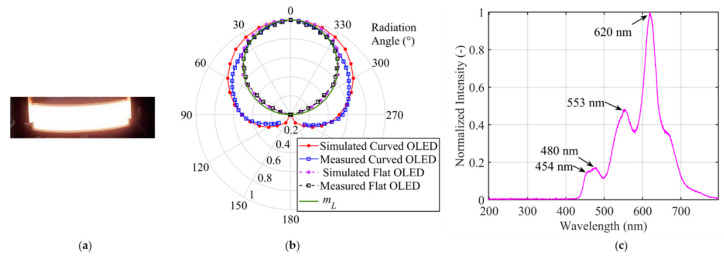
The flexible organic light emitting diodes (OLED) panel and its characteristics adopted in the simulation: (**a**) photograph, (**b**) the emission pattern of light source modeled for a flat and curved OLED, which is closely matched with the measured data, and (**c**) the normalized optical spectrum where the peak wavelengths are marked.

As inputs of the simulator, the measured characteristics of a flexible OLED from UNISAGA with a size of 200 × 50 mm^2^, see Figure 1a, were used. The measured beam pattern of the flexible OLED for flat and a half-circular configuration is depicted in Figure 1b, showing symmetry but not fitting with Lambertian radiation pattern (the solid line for *m_L_* = 1). A close match between the simulated and the measured beam patterns can be seen in Figure 1b. The measured spectrum profile of the flexible OLED is presented in Figure 1c, showing the red, green, and two blue components at 620, 553 and 454 and 480 nm, respectively.

### 2.2. Scenarios

**Figure 2:**
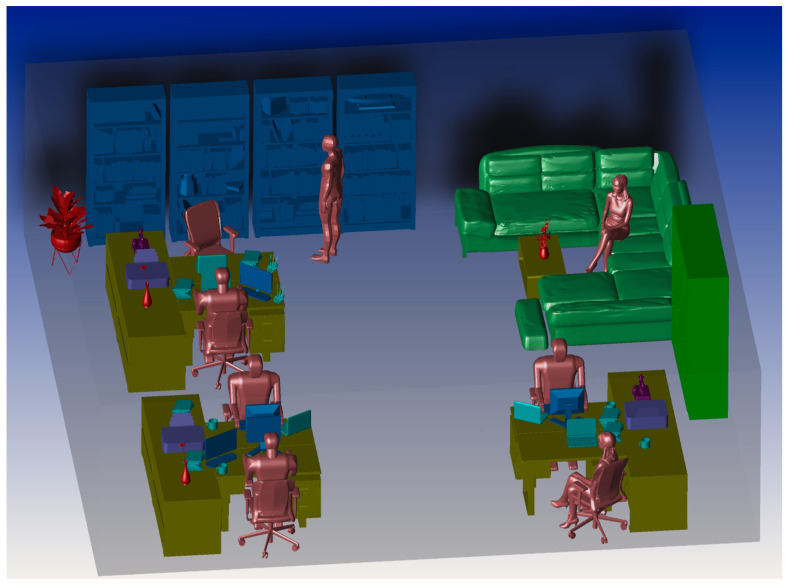
The three-dimensional office environment.

**Figure 3:**
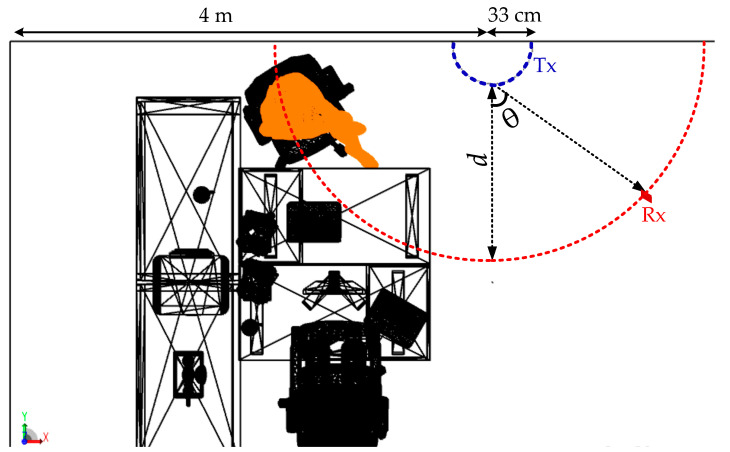
The proposed scenario which shows the location of receiver (Rx) and transmitter (Tx) giving a half-circular lighting.

Figure 2 shows the analyzed example of an office environment designed with the size of 10 × 10 × 3 m^3^ and a number of objects within. Here, a curved OLED with the size 1 × 0.5 m^2^ is mounted on the wall with a curvature radius of 32 cm. In the office environment, the scenario is to move the Rx over a semi-circular path, where the radius *d* is 2 m. An angle of radiation *θ* with respect to the normal from the center point of OLED (i.e., −90° < *θ* < 90°) is given, see Figure 3. The Rx height is assumed to be 1 m above the floor to represent people holding mobile phones while sitting at their desks. In simulation, we have not considered the synchronization. However, in real time systems synchronization protocols defined by the standards will be adopted, which does not affect the transmission characteristics of the proposed system.

**Figure 4:**
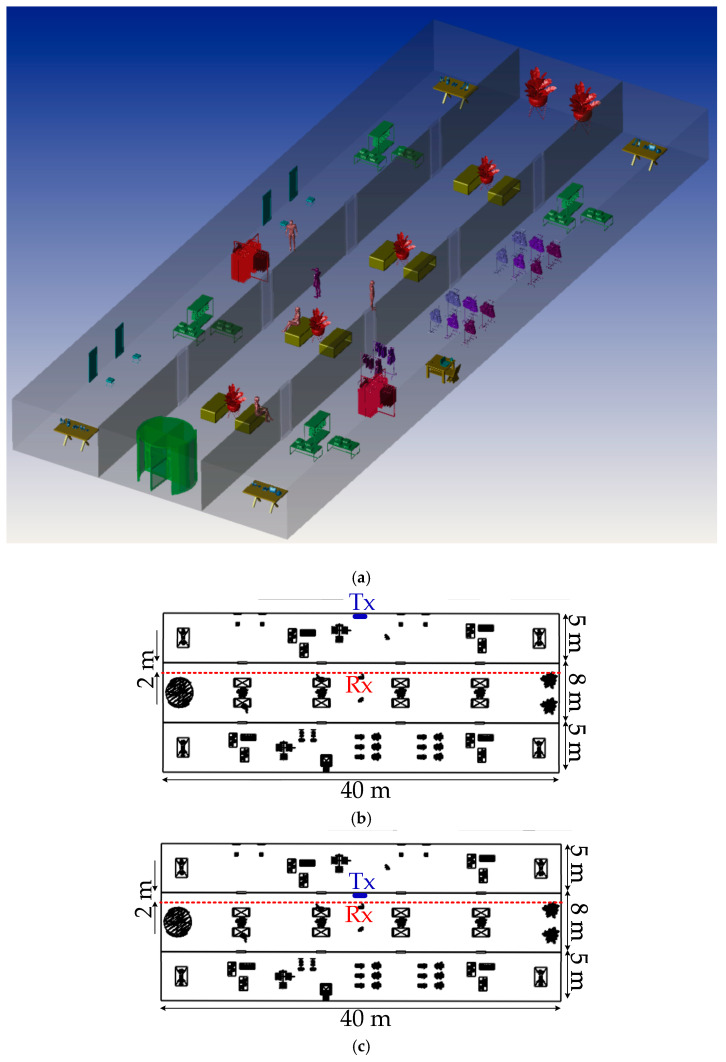
(**a**) The three-dimensional corridor environment and proposed scenarios which show the location of Rx and Tx, (**b**) case1 where Tx is located on inner shop wall behind window, and (**c**) case2 on wall or shop window inside the corridor.

**Figure 5:**
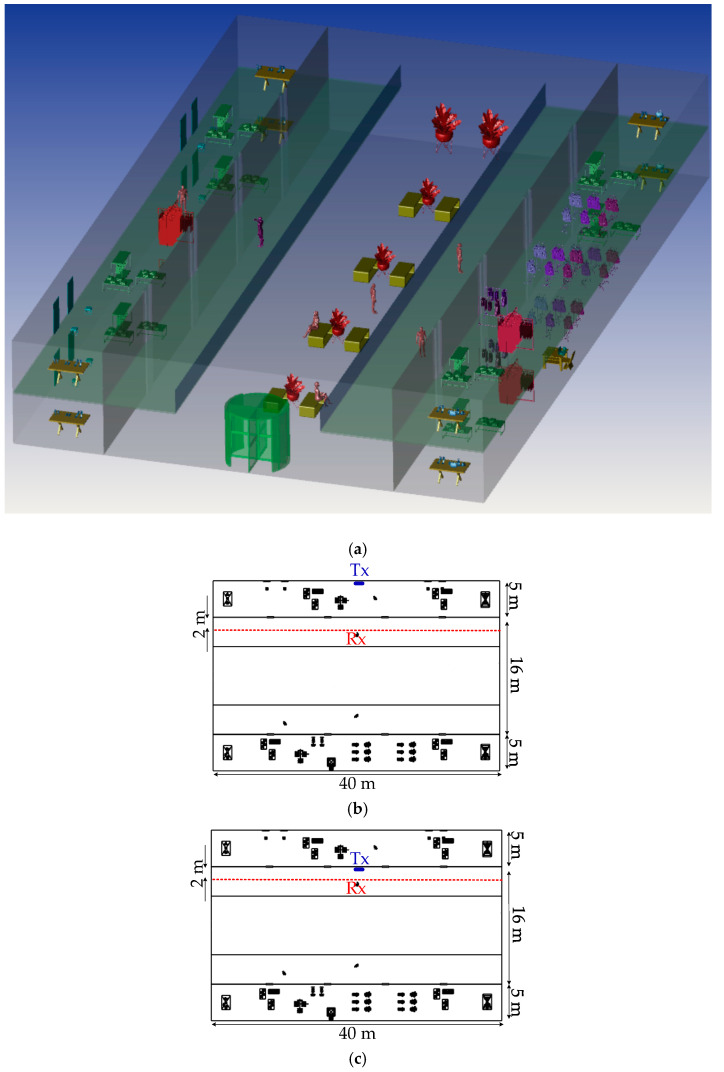
(**a**) The three-dimensional semi-open corridor environment and proposed scenarios which show the location of Rx and Tx, (**b**) where Tx is located on inner shop wall behind window (case1), and (**c**) on wall or shop window inside the corridor (case2).

Figure 4a and Figure 5a then show the 3D corridor and semi-open corridor environments inside a shopping mall, respectively, in which OLED acts as a light source. The semi-open corridor is typical especially for upper floors of the shopping mall, so we have chosen an example when the user walks on the first floor, having shop windows on one side and an open space on the other side. The user is moving on the straight path along the corridor (show by the red dashed-lines in Figure 4b,c and Figure 5b,c). The Rx is positioned at the height of 1.3 m above the floor level (i.e., the holding position of mobile by people). The user is moving along the shop windows at a distance of 2 m on the path donated as *d*_y_ from −20 to 20 m, where Tx is placed at 0 m position. For both empty and furnished corridor and semi-open environments, we have considered two scenarios of (case1), where the OLED panel is located on the inner shop wall behind the glass window and (case2) on the wall or shop window inside the corridor, see Figure 4b,c and Figure 5b,c. All the key system parameters adopted in this work, including the reflectance values of surfaces and the transmission coefficient of the glass windows, are given in Table 1.

## 3. Results

### 3.1. Comparison of Flat and Curved OLED Based System Performance

For intensity modulation/direct detection (IM/DD) optical transmission systems, the electrical SNR is defined as

(4) $$\mathrm{SNR}=\frac{{\left(\gamma P_{R}\right)}^{2}}{R_{b}N_{o}}=\frac{{\left(\gamma H(0)P_{E}\right)}^{2}}{R_{b}N_{o}},$$

where *γ* is the photodetector’s responsivity in (A/W), *P_E_* and *P_R_* are the emitted and received optical power, respectively, and *N*_0_/2 is double-sided power spectral density. Considering a link with non-return-to-zero (NRZ) on-off keying (OOK), the BER is given as [33]:

(5) $$\mathrm{BER}=\frac{1}{2}\mathrm{erfc}(\sqrt{\frac{\mathrm{SNR}}{2}}).$$

**Figure 6:**
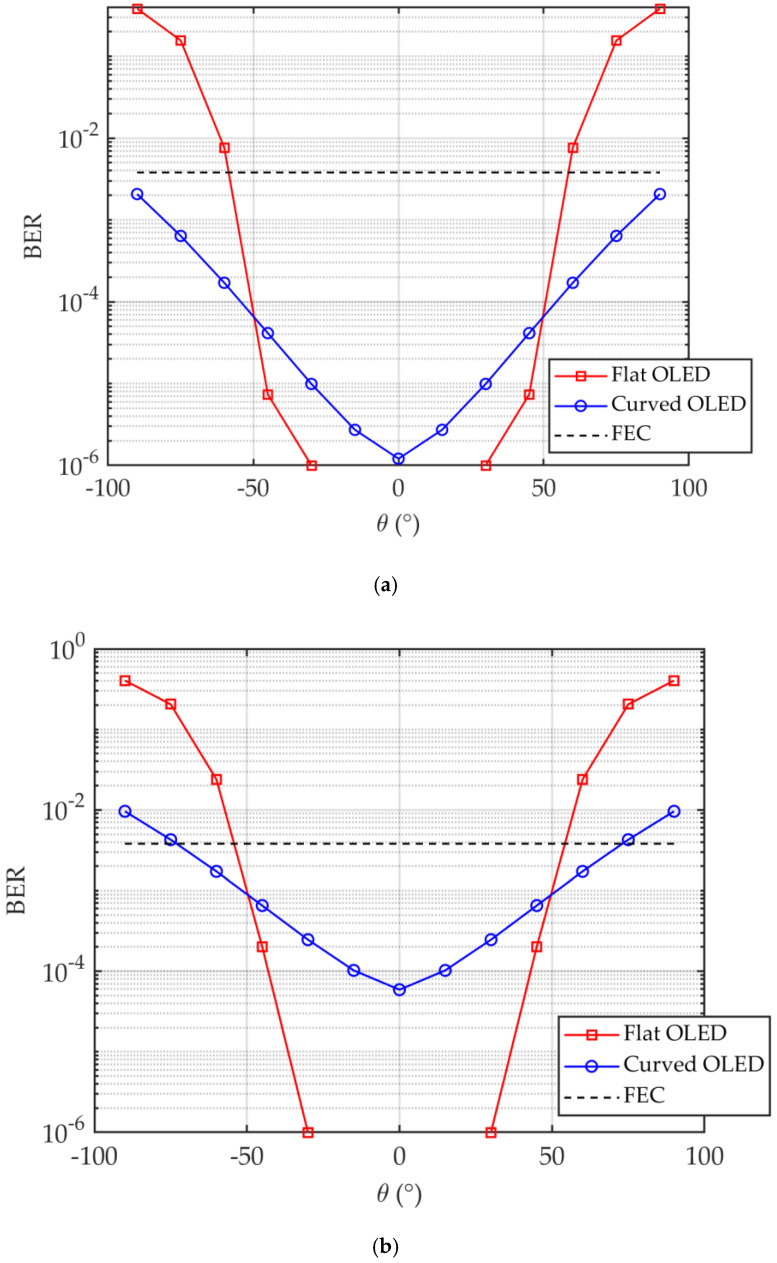
The bit-error-rate (BER) for flat and curved OLEDs at *R_b_* of: (**a**) 4 Mb/s, and (**b**) 6 Mb/s for an office.

Figure 6 shows the plots of the BER for flat and curved OLEDs at *R_b_* of 4 and 6 Mb/s along with the 7% forward error correction (FEC) BER limit of 3.8 × 10^−3^ [1] for an office. Note, for 4 Mb/s the BER is below the FEC limit for curved OLED. As illustrated, the BER plot displays a symmetry about the origin (i.e., at *θ* of 0°) because of the same achievable SNR that is maintained across the entire face of OLED. It is obvious that, for the curved OLED the BER is improved over a wider *θ* compared with the flat OLED. Note, for the flat OLED with −30° < *θ* < 30° the BER values are <10^−6^. At *R_b_* of 4 Mb/s, the BER remains below the FEC limit for *θ* within the range of ±90° and ±53° for the curved and flat OLEDs, respectively. However, for *R_b_* of 6 Mb/s, *θ* drops by 15° and 4° for the curved and flat OLEDs, respectively.

### 3.2. Channel Charactristics

The channel gain *H*(0) defines the achievable SNR for a given incident power. To quantify the data rate, *H*(0) and the optical signal attenuation OPL = −10log_10_(*H*(0)) caused by reflections and transmission in the free space are obtained [21,34]. The RMS delay spread is commonly used to define the time dispersion along the propagation path. The channel mean excess delay *τ* and the RMS delay spread *τ*_RMS_ are given as [27,31].

(6) $$\tau =\frac{\int_{0}^{\infty }t\times h(t)dt}{\int_{0}^{\infty }h(t)dt},$$

(7) $$\tau_{RMS}=\sqrt{\frac{\int_{0}^{\infty }{\left(t-\tau \right)}^{2}\times h(t)dt}{\int_{0}^{\infty }h(t)dt}}.$$

**Figure 7:**
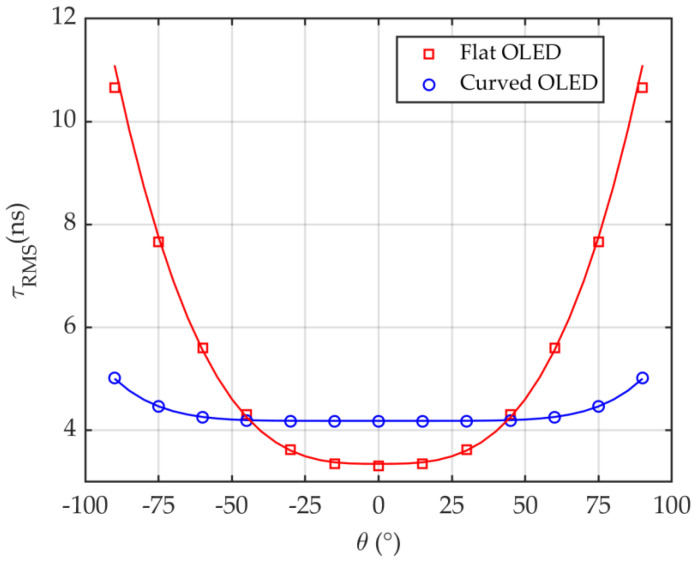
Comparison of a flat and curved OLEDs employed in the office in term of $\tau_{RMS}$.

Figure 7 depicts the *τ*_RMS_ plot for the flat and curved OLEDs in an office. The angle *θ* is shown in Figure 7 to identify the Rx’s location on the semi-circular path with the radius *d*. *τ*_RMS_ increases with *θ* reaching the maximum value of 5 and 10.7 ns at *θ* of 90° for the curved and flat OLEDs, respectively. It is obvious that, for the curved OLED, there is a slight change in *τ*_RMS_ by about 0.8 ns with respect to *θ*. However, *τ*_RMS_ has changed about 7.3 ns for the flat OLED. Note, there is a significant increase in *τ*_RMS_ for *θ* > 40° for the flat OLED. 

Using a non-linear approximation algorithm for both cases, a two-term power series model can be derived from simulations for τ _RMS_ as a function of *θ* given by

(8) $$\tau_{\mathrm{RMS}}=p_{1}\theta^{p_{2}}+p_{3},$$

where *p*_1_, *p*_2_ and *p*_3_ are summarized in Table 2. Note, the empirical parameters can vary based on the number of objects in the room and the size of the specified confined space.

**Figure 8:**
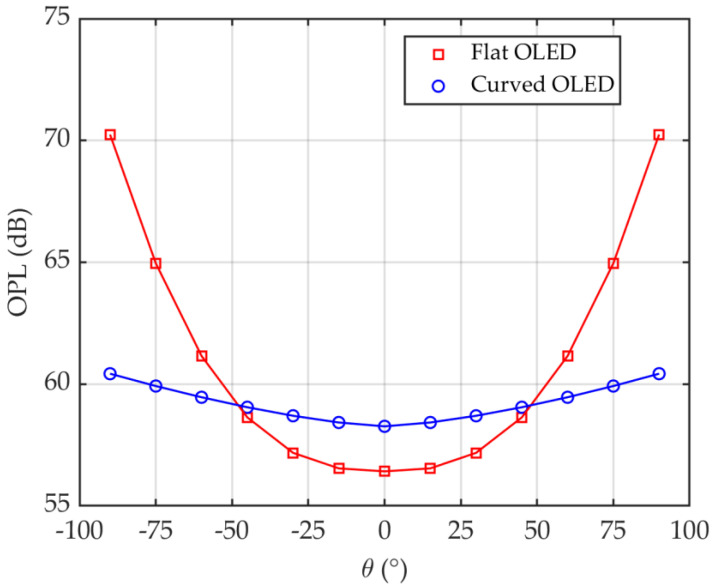
Comparison of a flat and curved OLEDs employed in the office in term of optical path loss (OPL).

Figure 8 shows the azimuthal dependence of the OPL distributions for flat and curved OLEDs for the proposed scenario in an office. OPL increases with *θ* reaching a maximum of 60.4 and 70.2 dB at *θ* of 90° for curved and flat OLEDs, respectively. Note, up to ~14 dB drop in the channel gain can be seen for the flat OLED when *θ* changes from 0° to 90°, which is considerably higher compared with the reduced ~2 dB channel gain for curved OLED. It can be seen that, for the flat OLED and *θ* < 30° there is an improvement in OPL by ~1.8 dB compared with the curved OLED. However, for *θ* > 45°, there is high received power enhancement for the curved OLED compared with the flat OLED, e.g., OPL penalties for flat OLED are 5 and 10 dB for *θ* of 75° and 90°, respectively. In addition, for both cases OPLs can be determined as the 2-term power series models as

(9) $$\mathrm{OPL}=a_{1}\theta^{a_{2}}+a_{3},$$

where the derived parameters *a*_1_, *a*_2_ and *a*_3_ are shown in Table 3.

**Figure 9:**
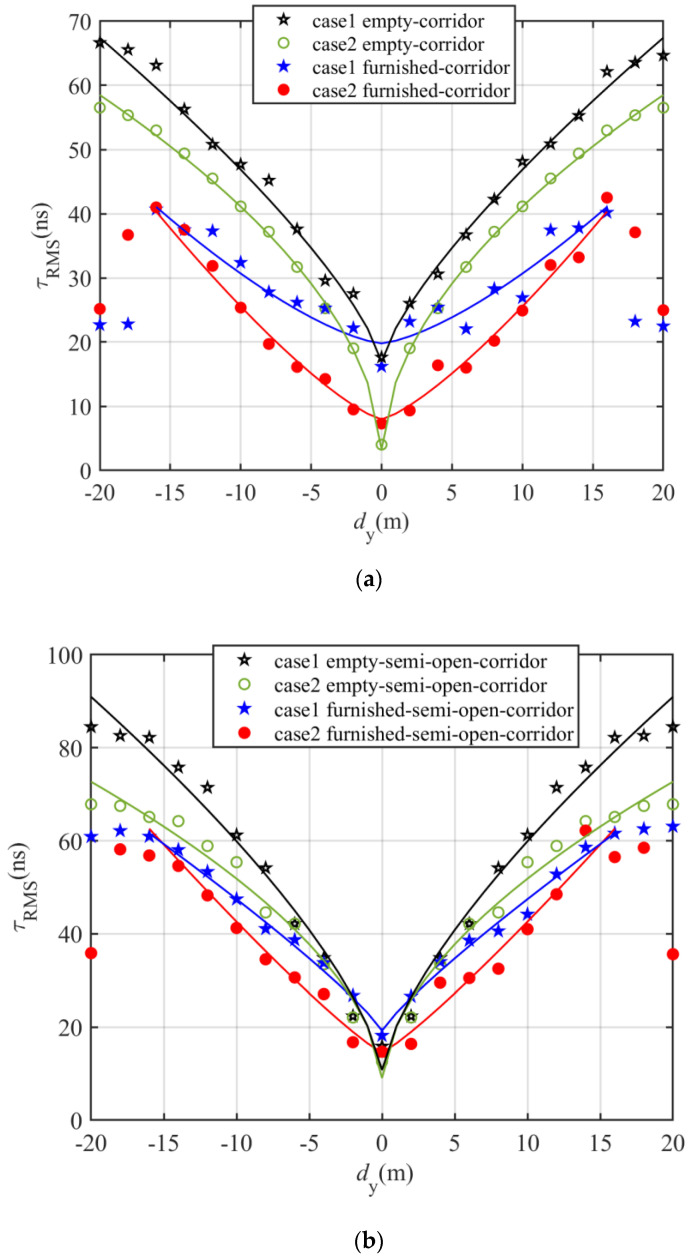
Channel characteristics in term of $\tau_{\mathrm{RMS}}$ for both: (**a**) corridor, and (**b**) semi-open corridor environment; when OLED are mounted on the inner shop wall behind the glass window (case1) and on the wall or shop window inside the corridor (case2) while the user is moving along the corridor.

**Figure 10:**
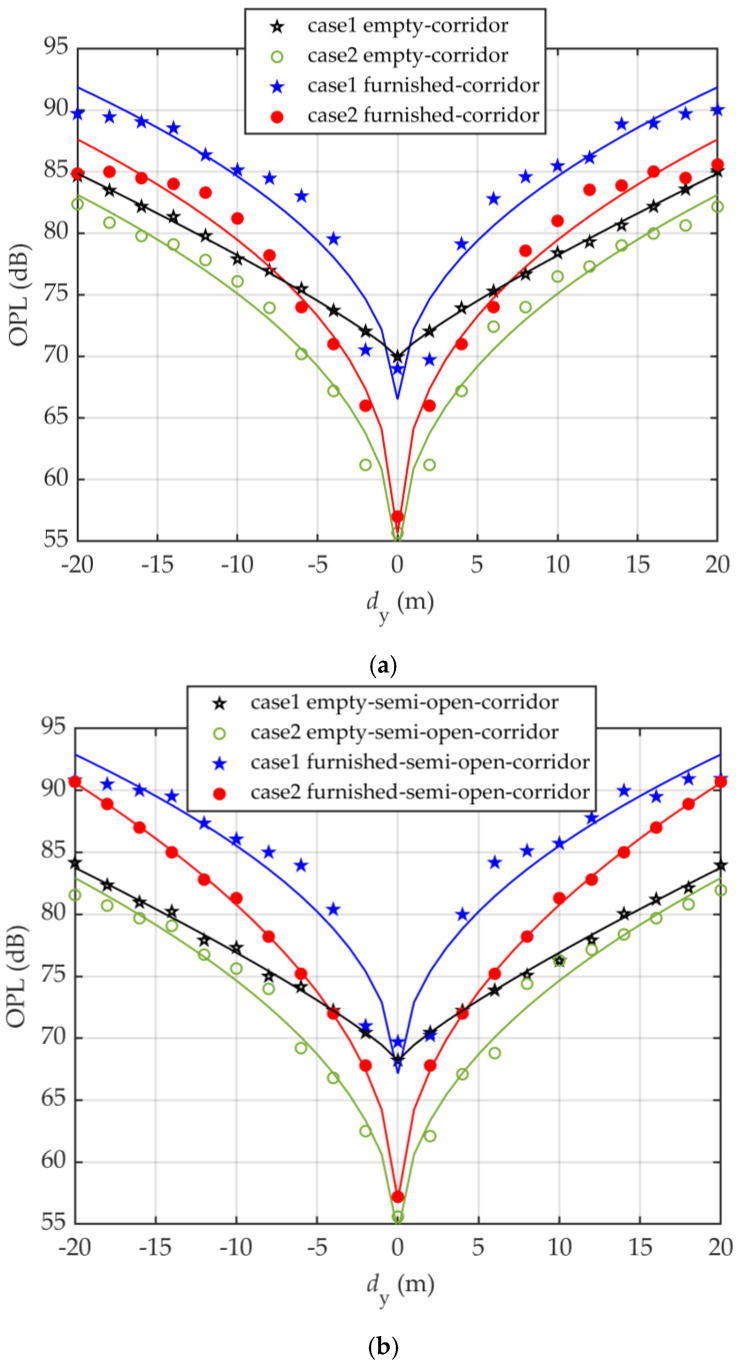
Channel characteristics in term of OPL for both: (**a**) corridor, and (**b**) semi-open corridor environment; when OLED are mounted on the inner shop wall behind the glass window (case1) and on the wall or shop window inside the corridor (case2) while the user is moving along the corridor.

Figure 9 and Figure 10 illustrate the channel characteristics for both open and semi-open corridor when OLED are mounted on the inner shop wall behind the glass window and on the wall or shop window inside the corridor while the user is moving along the corridor in terms of OPL and *τ*_RMS_. As can be seen, both OPL and *τ*_RMS_ plots show symmetry about the center of the indoor environment with minimum values at the center. Note, *τ*_RMS_ for furnished rooms is lower than the empty room for both corridor and semi open-corridor; e.g., in a semi-open corridor and case1, the *τ*_RMS_ values are 42.5 and 52.7 ns at 8 m and 52.2, 66.6 ns at 12 m for furnished and empty, respectively.

In furnished environments, *τ*_RMS_ increases with the distance reaching the maximum value of 40 and 60 ns at a distance of 16 m for the corridor and semi-open corridor, respectively. In all environments, *τ*_RMS_ drop significantly for case2 compared with case1. Note, in both furnished environments, there is a huge drop in *τ*_RMS_ for case2 compared with case1 for *d*_y_ up to 10 m; however, for *d*_y_ > 10 m *τ*_RMS_ for case2 reaches the corresponding value of case1. E.g., in a furnished corridor, for case2, *τ*_RMS_ values are lower than case1 by 13, 6 and 2 ns at *d*_y_ = 0, 8 and 14 m, respectively. However, in an empty corridor *τ*_RMS_ for case2 drop by 11, 5 and 5 ns at *d*_y_ = 0, 8 and 14 m, respectively.

Note, positioning OLEDs behind the window will result in decreased received optical power compare with when located on the outer-wall of shops inside the corridor; i.e., OPL for case2 is lower than case1 in all empty and furnished indoor environments. For instance, in a furnished semi-open corridor for case1, OPL penalties of 6.5, 4.8, 3.4, and 1.6 dB at 5, 10, 15, and 20 m, respectively, can be seen in comparison to case2. Additionally, for both cases, OPL in the corridor is lower compared with the semi-open corridor. e.g., in furnished environments and case2, OPL in the corridor reaches the maximum of 87.6 dB, which is lower than the value corresponding to the semi-open corridor (i.e., 90.7 dB). Note, the channel gain for both cases in furnished environments for *d*_y_ < 4 m remains the same; however, for case1 and *d*_y_ > 4 m, the OPL penalty of the furnished semi-open corridor is ~1 dB in contrary with the furnished corridor. For case2, there is a drop in OPL for the furnished corridor by 1.7 and 2.4 dB at *d*_y_ of longer distances of 10 and 15 m in comparison with the furnished semi-open corridor. 

In addition, the channel gain enhancement in an empty corridor in contrary with furnished one is 5 dB at 5 m reaching 7 dB at 15 m for case1. However, for case2 it remains around 4 dB for *d*_y_ > 4 m. In addition, the channel gain enhancement in an empty semi-open corridor in contrary with furnished one are 7.2 and 5 dB at 5 m increasing to 9.1 and 7 dB at 15 m for case1 and case2, respectively.

Using a non-linear approximation algorithm for both cases in all environment, a two-term power series model have been derived from simulations for τ_RMS_ and OPL as a function of *d*_y_ given by

(10) $$\tau_{\mathrm{RMS}}=r_{1}d_{y}{}^{r_{2}}+r_{3},$$

(11) $$\mathrm{OPL}=l_{1}d_{y}{}^{l_{2}}+l_{3},$$

where the derived values of *r*_1_, *r*_2_, *r*_3_, *l*_1_, *l*_2_, and *l*_3_ are summarized in Table 4 and Table 5.

## 4. Conclusions

In this paper, we investigated the performance of OLED-based VLC system and the channel characteristics in office, corridor, and semi-open environments. The measured beam pattern profile of the curved OLED was closely matched with the simulation result. We showed that, when a flat OLED was used in an office, *τ*_RMS_ increased significantly by 7.3 ns compared with 1 ns for the curved OLED. In the office, contrary to the flat OLED, the curved OLED showed improved BER performance over a wider range of *θ*. A data rate of 4 Mb/s was achieved using both the curved and flat OLEDs for *θ* within the range of ±90° and ±53°, respectively. A two-term power series model was found to match *τ*_RMS_ and OPL as a function of *θ* and *d*_y_ for the office and corridors, respectively and models’ parameters for all three environments with and without furniture were derived. We showed that, when OLED is positioned on the outer wall of shops inside the corridor, the channel gain enhanced in contrast to them being located on inner shop wall, e.g., the channel gain enhanced by 5.2 and 4.8 dB at 10 m for furnished corridor and semi-open corridor, respectively. Moreover, the channel gain in the corridor was higher compared with the semi-open corridor. As a result, for case2, there was an enhancement in the channel gain for the furnished corridor by 1.7 and 2.4 dB at *d*_y_ of 10 and 15 m in comparison with the furnished semi-open corridor. 

## Figures and Tables

**Table 1:** The system parameters.

Item	Parameter	Value		
Surface material refractivity in % (RGB)	Chair, sofa (leather)	24	18.8	16.3
	Coffee cup (ceramics)	97.1	96.2	92.3
	Human clothes (cotton)	67	58	45.6
	Plant (leaf)	14	5.9	8.2
	Desk, book shelf, book (pine wood)	70	51	33.1
	Laptop, PC, printer, and telephone (black gloss paint)	3.4	3.2	3.2
Transmission coefficient in % (RGB)	Glass windows	88	90	87
Room size	Office	10 × 10 × 3 m^3^		
	Corridor	18 × 40 × 3 m^3^		
	Semi-open corridor	26 × 40 × 3 m^3^		
Tx	Dimension	1 × 0.5 m^2^		
	Type	Flexible		
	Bandwidth	50 kHz		
	Power of lighting	10 W		
	Number of OLED panels	19		
	Number of chip/LED panel	64		
	Power of each chip	8.2 mW		
	Curvature radius	32 cm		
	Location in office	(4, 0.33, 1.5) m		
	Location in corridor and semi-open corridor	case1: (0.1, 20, 1.5) m case2: (4.9, 20, 1.5) m		
Channel	Time resolution	0.2 ns		
Rx	Active area of PD	1 cm^2^		
	Responsivity	0.4 A/W		
	FOV	90°		
	Incident angle	0°		
	One sided noise power spectral density *N*_o_	10^−19^ W/Hz		

**Table 2:** Numerical modeling parameters for τ_RMS_ in the case of using flat and curved OLEDs in office.

OLED Type	*p*_1_	*p*_2_	*p*_3_
Curved	8.707 × 10^−10^	4.589	4.172
Flat	1.765 × 10^−5^	2.875	3.312

**Table 3:** Numerical modeling parameters for OPL in the case of using flat and curved OLEDs in office.

OLED Type	*a*_1_	*a*_2_	*a*_3_
Curved	0.2964 × 10^−2^	1.465	58.26
Flat	9.315 × 10^−5^	2.646	56.42

**Table 4:** Numerical modeling parameters for τ_RMS_ in both corridor and semi-open corridor.

Environment	*r*_1_	*r*_2_	*r*_3_
case1 empty-corridor	5.32	0.7519	16.83
case2 empty-corridor	11.26	0.5358	2.446
case1 furnished-corridor	0.409	1.424	19.79
case2 furnished-corridor	0.890	1.296	7.947
case1 empty-semi-open corridor	9.777	0.7035	10.5
case2 empty-semi-open corridor	11.9	0.5637	8.267
case1 furnished-semi-open corridor	3.860	0.864	19.214
case2 furnished-semi-open corridor	2.020	1.143	14.513

**Table 5:** Numerical modeling parameters for OPL in both corridor and semi-open corridor.

Environment	*l*_1_	*l*_2_	*l*_3_
case1 empty-corridor	1.160	0.852	69.95
case2 empty-corridor	7.893	0.446	53.01
case1 furnished-corridor	6.379	0.469	65.823
case2 furnished-corridor	10.010	0.403	54.130
case1 empty-semi-open corridor	1.265	0.838	68.190
case2 empty-semi-open corridor	6.946	0.480	53.670
case1 furnished-semi-open corridor	6.453	0.470	66.451
case2 furnished-semi-open corridor	8.039	0.485	56.180
